# High‐Rate Organic Cathode Constructed by Iron‐Hexaazatrinaphthalene Tricarboxylic Acid Coordination Polymer for Li‐Ion Batteries

**DOI:** 10.1002/advs.202205069

**Published:** 2022-11-10

**Authors:** Yifan Wang, Zelong Qiao, Kexin Liu, Le Yu, Yingying Lv, Liyi Shi, Yin Zhao, Dapeng Cao, Zhuyi Wang, Shitao Wang, Shuai Yuan

**Affiliations:** ^1^ School of Materials Science and Engineering Shanghai University Shanghai 200444 P. R. China; ^2^ Research Centre of Nanoscience and Nanotechnology Shanghai University Shanghai 200444 P. R. China; ^3^ State Key Lab of Organic‐Inorganic Composites Beijing University of Chemical Technology Beijing 100029 P. R. China; ^4^ Emerging Industries Institute Shanghai University Jiaxing Zhejiang 314006 P. R. China

**Keywords:** ion transport, Li‐ion batteries, metal‐organic coordination polymer, organic cathode, porous structure

## Abstract

The sluggish ion‐transport in electrodes and low utilization of active materials are critical limitations of organic cathodes, which lead to the slow reaction dynamics and low specific capacity. In this study, the hierarchical tube is constructed by iron‐hexaazatrinaphthalene tricarboxylic acid coordination polymer (Fe‐HATNTA), using HATNTA as the self‐engaged template to coordinate with Fe^2+^ ions. This Fe‐HATNTA tube with hierarchical porous structure ensures the sufficient contact between electrolyte and active materials, shortens the diffusion distance, and provides more favorable transport pathways for ions. When employed as the cathode for rechargeable Li‐ion batteries, Fe‐HATNTA delivers a high specific capacity (244 mAh g^−1^ at 50 mA g^−1^, 91% of theoretical capacity), excellent rate capability (128 mAh g^−1^ at 9 A g^−1^), and a long‐term cycle life (73.9% retention over 3000 cycles at 5 A g^−1^). Moreover, the Li^+^ ions storage and conduction mechanisms are further disclosed by the ex situ and in situ characterizations, kinetic analyses, and theoretical calculations. This work is expected to boost further enthusiasm for developing the hierarchical structured metal‐organic coordination polymers with superb ionic storage and transport as high‐performance organic cathodes.

## Graphical abstract

The iron‐hexaazatrinaphthalene tricarboxylic acid coordination polymer (Fe‐HATNTA) tube is prepared by using HATNTA as the self‐engaged template to coordinate with Fe^2+^ ions. The narrow LUMO–HOMO gap, low diffusion barriers, and hierarchically porous structure facilitate the electrons and Li^+^ ions transport. Fe‐HATNTA delivers a capacity of 244 mAh g^−1^ at 50 mA g^−1^ and 128 mAh g^−1^ at 9 A g^−1^.

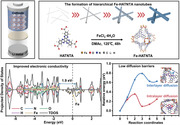

## 1 Introduction

Over the past decade, driven by the development of electric vehicles, energy‐storage systems, and other energy fields, Li‐ion batteries (LIBs) have urgent needs for both increasing energy density and fast charging/discharging performance.^[^ ^1^, ^2^, ^3^ ^]^ The energy density of a battery mainly depends on the cathode and anode materials. However, most commonly conventional cathodes such as transition metal oxides, are still facing severe challenges such as limited theoretical specific capacity, resource scarcity, high cost, and environmental problems.^[^ ^4^, ^5^, ^6^ ^]^

Fortunately, there are kinds of organic materials with high theoretical capacities, owing to the ample redox‐actives and nonintercalation redox mechanism.^[^ ^4^, ^6^ ^]^ For example, hexaazatrinaphthylene (HATN) based on imines (418 mAh g^−1^),^[^ ^7^ ^]^ *p*‐benzoquinone (BQ) (496 mAh g^−1^),^[^ ^8^ ^]^ anthraquinone (AQ) (257 mAh g^−1^)^[^ ^5^ ^]^ have shown high capacities. Coupled with the material abundance, cost‐effectiveness, eco‐friendliness, sustainability, and tunable molecular structures, organic materials have become a promising alternative to cathodes.^[^ ^9^, ^10^, ^11^ ^]^ However, one of the greatest challenges for these organic cathodes is the inferior rate performance, particularly at high current densities, which is mainly caused by the slow ions diffusion and poor electrons transfer within the bulk electrode.^[^ ^3^, ^12^, ^13^, ^14^, ^15^ ^]^

Recently, the metal‐organic coordination polymers (MOCPs) have emerged as one kind of promising organic candidate to overcome the above challenges. Since MOCPs are composed of transition metal ions and organic ligands,^[^ ^16^, ^17^ ^]^ their energy level structures and the energy gaps can be regulated by appropriately choosing the ligands and metal ions. According to previous reports, the electronic conductivity of organic materials can be enhanced by reducing the HOMO–LUMO energy gap,^[^ ^18^ ^]^ while the ion conduction may be facilitated by producing porous organic materials.^[^ ^13^ ^]^ Therefore, porous MOCPs are expected to be used as high‐performance organic cathode materials. In our previous work,^[^ ^19^ ^]^ we selected hexaazatriphenylene hexacarbonitrile (HAT‐CN), a cyano‐group‐modified HATN derivative, as the ligand to synthesize the Co‐(HAT‐CN) composite as a modified MOCP with a narrowed LUMO‐HOMO gap of 0.61 eV for an improved electronic conductivity. However, most porous structures of the current MOCPs are still of micropore, which limits the transport of ions and utilization of active sites.^[^ ^3^, ^20^, ^21^, ^22^, ^23^ ^]^ If hierarchical pore structures composed of micropore and meso/macropore could be suitably designed and fabricated for MOCPs, the fast ion transport and increased accessible active sites would be available to realize excellent rate capability and large specific capacity.

Herein, an iron‐hexaazatrinaphthalene tricarboxylic acid (Fe‐HATNTA) MOCP with hierarchically porous tube structure was successfully synthesized as a promising LIB cathode, through a self‐engaged coordination reaction between Fe^2+^ ions and rod‐like HATNTA template. The hollow tube with the unique integration of micropores and mesopores ensures abundant surface areas to facilitate the electrolyte/electrode contact and shortens the diffusion distance of Li^+^ ions. In addition, the hollow structure of tube can provide highway for accelerating transport of Li^+^ ions. Meanwhile, Fe^2+^ ions play an important role in reducing the HOMO–LUMO gap of Fe‐HATNTA for enhanced electronic conductivity. As expected, Fe‐HATNTA cathode delivers a large specific capacity of 244 mAh g^−1^ at 50 mA g^−1^, a large high‐rate capability (128 mAh g^−1^ at 9 A g^−1^), and a long‐term cycle life over 3000 cycles at 5A g^−1^. Moreover, X‐ray photoelectron spectroscopy (XPS) and in situ Raman spectra, kinetic analyses, and theoretical calculations are also conjointly conducted to further disclose the internal mechanism for high electrochemical performance.

## 2 Results and Discussion

### 2.1 Synthesis and Characterization of Iron‐Hexaazatrinaphthalene Tricarboxylic Acid

**Scheme 1:**
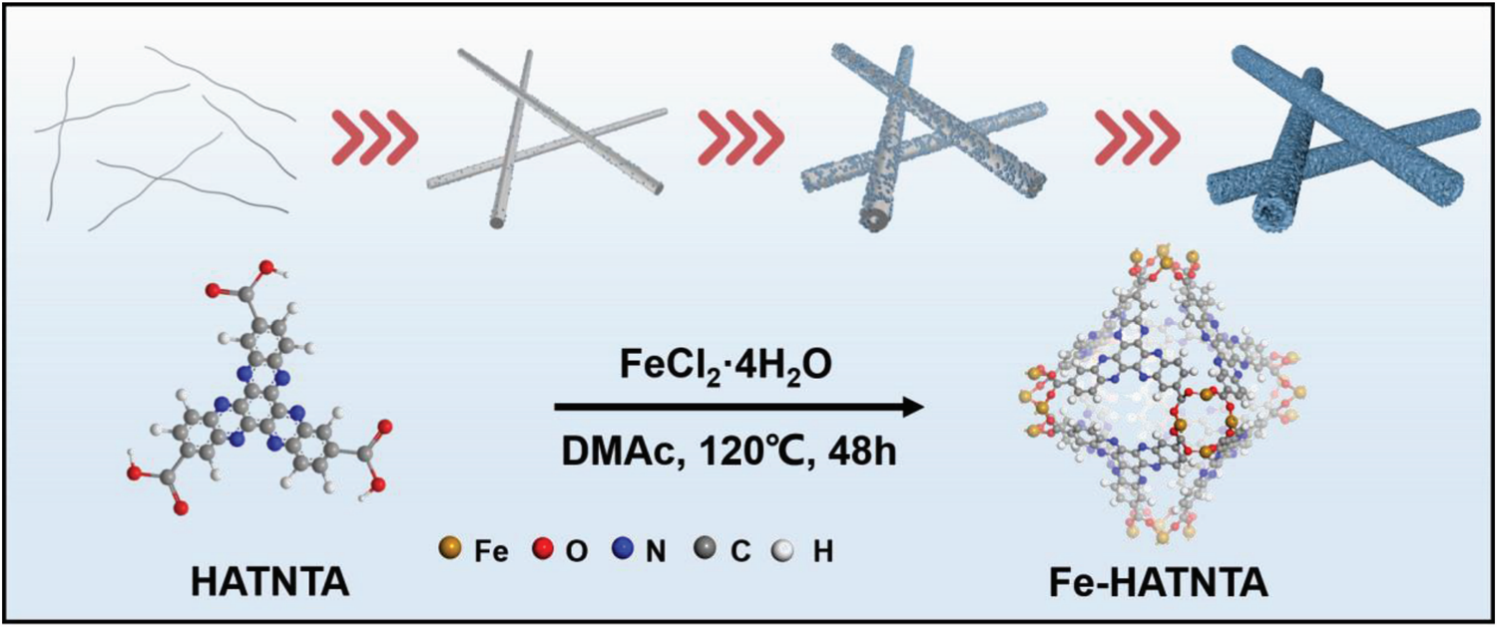
The formation process of Fe‐HATNTA MOCP with tube‐like structure using rod‐like HATNTA as the self‐engaged template.

Fe‐HATNTA is synthesized from the solvothermal reaction between HATNTA monomers and Fe (II) salts (**Scheme** **1**). The as‐obtained MOCP contains abundant C = N redox‐active groups derived from HATNTA monomers, and well‐coordinated building blocks. Besides, this MOCP framework is found to be a hollow tube with hierarchical porosity. The specific process is that HATNTA fibers self‐assemble in the solvent to form a nanorod before coordination, and then the rod‐like HATNTA works as a sacrificial template to coordinate with the iron sources.

**Figure 1:**
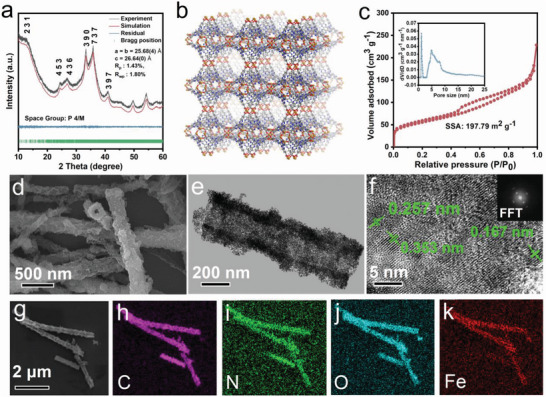
Structural and morphological analysis of Fe‐HATNTA. a) Rietveld refined XRD patterns. b) The simulated 3D network of Fe‐HATNTA. c) N_2_ sorption isotherms of Fe‐HATNTA. Inset: Pore size distribution obtained from the N_2_ isotherm. d) FESEM image and e) HRTEM image of Fe‐HATNTA. f) HRTEM lattice image and the related FFT pattern, scale bar: 5 nm. g) FESEM and the corresponding EDS mapping images for h) C, i) N, j) O, and k) Fe of Fe‐HATNTA.

As shown in **Figure** **1**a, the powder X‐ray diffraction (PXRD) pattern of Fe‐HATNTA (black curve) exhibits prominent peaks at intense peaks at 12.7°, 24.4°, 26.8°, 33.3°, 35.9°, and 40.9°, which can be assigned to the (231), (453), (436), (390), (737), and (397) planes. In addition, the XRD pattern of Fe‐HATNTA is distinctly different from that of HATNTA and self‐assembled HATNTA (Figure S1, Supporting Information). The characteristic peaks of Fe‐HATNTA are not observed in HATNTA and self‐assembled HATNTA, indicating an obvious difference in structure. Moreover, the refined PXRD pattern of Fe‐HATNTA by Pawley refinement also fits well with experimental data with negligible difference. The refined unit cell parameters for Fe‐HATNTA are obtained as *a = b* = 25.684 Å, *c* = 26.640 Å, and *α = β = γ* = 90° (residuals *R*_p_ of 1.43% and *R*_wp_ of 1.80%). Based on the PXRD results, it is determined that Fe‐HATNTA possesses a partially augmented the topology with P4/M space group^[^ ^24^ ^]^ (Figure 1b).

Brunauer‐Emmett‐Teller (BET) surface area and pore size distribution of Fe‐HATNTA are investigated by the nitrogen adsorption‐desorption isotherms. As displayed in Figure 1c, BET specific surface area of Fe‐HATNTA is calculated to be 197.79 m^2^ g^−1^, much higher than that of HATNTA (36.59 m^2^ g^−1^) (Figure S2, Supporting Information). The pore size distribution at 0.9–1.8 nm refers to the micropores of Fe‐HATNTA framework, while the mesopores centered at 3–10 nm are constructed by accumulation of ultrafine nanoparticles. The pore size of micropores is close to the calculated value (≈1.6 nm), further validating the simulated structure. The high‐resolution transmission electron microscopy (HRTEM) images show the mesopores are evenly dispersed throughout the materials (Figure S3, Supporting Information). These rich hierarchical pores are highly conducive for Li^+^ ions to fast infiltrate into the interior redox sites, thereby enhancing the redox dynamics.

The morphology of Fe‐HATNTA is further characterized by field‐emission scanning electron microscopy (FESEM). The FESEM images illustrate the tube‐like Fe‐HATNTA nanostructure, which is assembled by numerous nanocrystals (Figure 1d and Figure S4, Supporting Information). The transmission electron microscopy (TEM) image (Figure 1e) also reveals the tube has a diameter in the range of 300–400 nm and a shell thickness of 100–150 nm. Moreover, the formation mechanism of the tube‐like nanostructure is further revealed by investigating the growth process. The varied morphologies at different reaction stages are characterized via FESEM and TEM observations (Figure S5, Supporting Information). At the initial stage of the reaction, driven by a combination of the non‐covalent interactions such as hydrogen bonding and *π*–*π* stacking in the solution, HATNTA molecules assemble into a fibrous structure^[^ ^25^, ^26^ ^]^ (Figure S5a,e, Supporting Information). As the reaction time is further prolonged, iron ions begin to coordinate with HTANTA to form Fe‐HATNTA nanocrystals epitaxially growing on the surface of HATNTA nanorod assemblies (Figure S5b,f, Supporting Information). In this stage, Fe‐HATNTA nanocrystals keep the growth on the surface of HATNTA nanorod‐shaped assemblies, while HATNTA rod‐shaped assemblies are continuously consumed (Figure S5c,g, Supporting Information). At the end of the reaction, the rod‐shaped HATNTA reactant is completely consumed to generate hierarchical Fe‐HATNTA tubes consisting of numerous nanoparticles (Figure S5d,h, Supporting Information). Based on these results, it can be concluded that the porous Fe‐HATNTA tubes are formed through the template‐engaged method using HATNTA nanorod as a sacrificial scaffold to coordinate with Fe^2+^ ions.

In addition, HRTEM analysis reveals distinct lattice fringes with the *d*‐spacing of 0.257, 0.353, and 0.167 nm (Figure 1f), which agrees well with the PXRD pattern. At the same time, the inset fast Fourier transform pattern in further confirms the good crystallinity of Fe‐HATNTA (Figure 1f). The energy dispersive spectroscopy (EDS) elemental mapping results (Figure 1g–k) show the homogeneous distribution of Fe, O, N, and C in Fe‐HATNTA. Fe‐HATNTA tubes with hierarchical porosity can offer an ideal system to favor the efficient utilization of the redox‐active units and fast diffusion of ions.

Fourier transformed infrared (FTIR) characterization further reveals the molecular structure of Fe‐HATNTA (Figure S6, Supporting Information). The signals at 3400–2700 cm^−1^ are contributed from the stretching vibration of —OH bond,^[^ ^27^ ^]^ which are clearly weakened in Fe‐HATNTA. The absorption peak at 1710 cm^−1^, ascribed to the stretching vibrations of anti‐symmetrical —COO^−^ group, shifts to 1561 cm^−1^. Meanwhile, symmetrical stretching vibrations of —COO^−^ group at 1377 cm^−1^ are unambiguously strengthened.^[^ ^28^, ^29^ ^]^ At the same time, the two characteristic stretching vibrations of C = N (1520 cm^−1^) and C—N (1403 cm^−1^) are still preserved in the Raman spectrum of Fe‐HATNTA (Figure S7, Supporting Information).^[^ ^30^, ^31^, ^32^ ^]^ These results indicate that carboxyl group successfully coordinates with metal ions.

### 2.2 Electrochemical Performance of Iron‐Hexaazatrinaphthalene Tricarboxylic Acid

**Figure 2:**
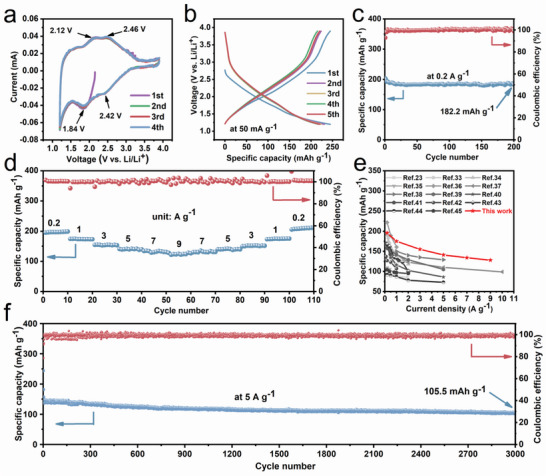
Electrochemical performance of Fe‐HATNTA. a) CV curves at the scan rate of 0.5 mV s^−1^. b) Charge‐discharge curves at the current density of 50 mA g^−1^. c) Cycling performance at 0.2 A g^−1^. d) Rate performance at various current densities. e) Comparison of the rate performance between this work and previously reported representative organic cathode materials. f) Cycling performance at 5 A g^−1^.

The electrochemical behavior for Fe‐HATNTA cathode is then performed using cyclic voltammogram (CV) in a voltage range of 1.2–3.9 V with a scan rate of 0.5 mV s^−1^ (**Figure** **2**a). There are two redox couples stabilized at ≈2.42 V and 1.84 V, in accordance with the proposed two‐step three‐electron transfer mechanism (Figure S8, Supporting Information).^[^ ^7^ ^]^ The ensuing CV curves largely coincide with each other, indicating the highly stable and reversible nature of Fe‐HATNTA during the redox reaction. Figure 2b shows a high reversible capacity of about 244 mAh g^−1^ at 50 mA g^−1^ (91% of the theoretical capacity), along with a high initial coulombic efficiency of around 97%, and the subsequent curves after the initial cycle are also overlapped. Besides, Fe‐HATNTA is capable of stable cycling as well (Figure 2c). When cycled at 200 mA g^−1^, it achieves a remarkably large specific capacity of 182.2 mAh g^−1^ over 200 cycles with a high capacity retention of 89.5%. The UV–vis spectra then indicate the outstanding insolubility of Fe‐HATNTA in electrolytes (Figure S9, Supporting Information), which contributing to the boosted electro‐chemical performance.

The rate capability of Fe‐HATNTA is evaluated at the progressively increased current levels. Figure 2d shows stable reversible capacities of 195.5, 174.6, 154.7, 140.8, and 134 mAh g^−1^ at according current rates of 0.2, 1, 3, 5, and 7 A g^−1^. Impressively, even at a very high current density of 9 A g^−1^, Fe‐HATNTA cathode still demonstrates a reversible capacity as high as 128 mAh g^−1^, corresponding to 65.5% of the capacity at 0.2 A g^−1^. Moreover, as each current level is symmetrically reverted, so does each corresponding capacity. It can be seen that Fe‐HATNTA presents a preeminent tolerance to the back‐and‐forth deep cycles. Figure S10, Supporting Information, displays the charge/discharge curves of Fe‐HATNTA at various current densities from 0.2 to 6 A g^−1^. Encouragingly, such a good rate performance clearly outperforms HATNTA cathode (Figure S11a,b, Supporting Information) and many other organic cathode materials in previously related reports (Figure 2e).^[^ ^23^, ^33^, ^34^, ^35^, ^36^, ^37^, ^38^, ^39^, ^40^, ^41^, ^42^, ^43^, ^44^ ^]^ Figure 2f shows a long‐term cycling performance at the high current density of 5 A g^−1^. The reversible capacity of 105.5 mAh g^−1^ is delivered at the end of 3000 cycles with ≈73.9% capacity retention.

Considering the theoretically adverse effect of inactive materials on Li^+^ ions transport, we further explore the feasibility with higher content of active material (the ratio of active material: carbon black: polyvinylidene fluoride is increased from 6:3:1 to 8:1:1). When cycled at 200 mA g^−1^, the capacity still sustains at 127.9 mAh g^−1^ over 200 cycles, yielding a capacity retention of 89.3% (Figure S12b, Supporting Information). The corresponding charge/discharge curves are highly overlapped as shown in Figure S12a, Supporting Information. It should be mentioned that the rate capability is also striking with high ratio of active material, demonstrating a capacity of 112.1 mAh g^−1^ at 2 A g^−1^ (Figure S12c,d, Supporting Information). These results thereby point to a significant potential of Fe‐HATNTA for practical application.

### 2.3 In Situ and Ex Situ Studies on the Structural Analysis

**Figure 3:**
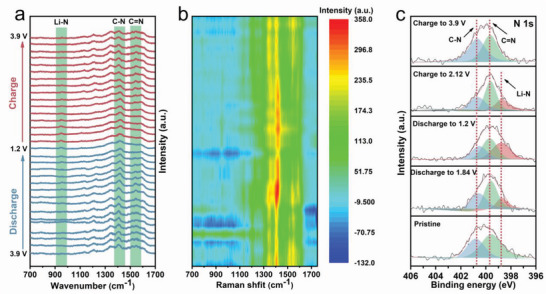
The structural evolution during the charge/discharge. a) In situ Raman spectra of Fe‐HATNTA cathode in the charging‐discharging process. b) The color‐mapped profile for in situ Raman spectra in different charge/discharge states. c) XPS spectra of high‐resolution N 1s at different electrochemical states.

To monitor the structural evolution and reversibility of Fe‐HATNTA electrode during the charge/discharge process, in situ Raman tests are carried out. As shown in **Figure** **3**a,b, in situ Raman spectra and the color‐mapped profile exhibit the peaks at 1534 and 1403 cm^−1^ in the initial state, assigned to C = N and C—N groups, respectively.^[^ ^30^, ^31^, ^32^ ^]^ Nevertheless, the intensity of C = N peak gradually weakens, while the intensity of C—N peak increases in the discharging process. In the charging process, C = N peak and C—N peak show the adverse trends. At the same time, a peak belonging to Li—N emerges at ≈950 cm^−1^ gradually rises and falls in intensity during the lithiation and the de‐lithiation processes, respectively. These results demonstrate that lithiation and de‐lithiation with C = N redox‐active sites are highly reversible in the Fe‐HATNA electrode. The structural evolution is also evidenced and supported by XPS. The high‐resolution N 1s spectrum in the pristine electrode shows two peaks at 400.7 and 399.6 eV, attributed to C—N and C = N, respectively (Figure 3c).^[^ ^20^ ^]^ When discharging from the pristine state to a fully discharged state (1.2 V), the peak for C = N is gradually decreased, while a new peak at 398.7 eV that is ascribed to Li—N bond emerges and gradually increases with discharge process, implying that C = N acts as a redox active site to uptake Li^+^ ion during the lithiation process. After recharged to 3.9 V from the fully discharge state, the restoration of C = N peak and the disappearance of Li‐N are observed at the fully charged state. These data indicate the reversible intercalation/de‐intercalation of Li^+^ ion in Fe‐HATNTA. As for the XPS spectra of O 1s region, the peaks centered ≈at 532.7 and 532.1 eV are assigned to C—O and C = O, respectively (Figure S13, Supporting Information).^[^ ^29^ ^]^ No obvious change occurs in the different charge states, pointing to the noninvolvement of —COO^−^ groups during the redox reaction. In the Fe 2p XPS spectra (Figures S14 and S15, Supporting Information), the deconvoluted peaks, centered at 709.9 and 715.8 eV, are attributed to Fe 2p_3/2_ and its satellite,^[^ ^28^ ^]^ respectively, which display negligible change in cycling process. Moreover, according to the electron paramagnetic resonance spectra (Figure S16), there is no Fe^3+^ ions peak in the different charge states. That is to say, the metal ions serve only as the bodyguard to stabilize Fe‐HATNTA structure rather than participant in redox reaction. Based on these results, it is concluded that only C = N undergoes reversible redox reactions, and the N atoms are bonded to Li^+^ ions during the lithiation and de‐lithiation process.

### 2.4 Electrode Kinetics

**Figure 4:**
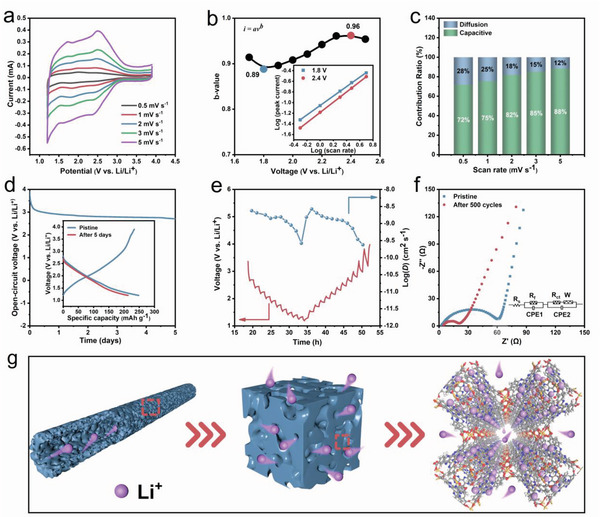
Electrode kinetics of Fe‐HATNTA. a) CV curves of Fe‐HATNTA at various scan rates. b) *b*‐values for Fe‐HATNTA electrodes plotted as a function of potential for cathodic sweeps. Inset shows *b*‐value fitted results at 1.8 V (blue, *b* = 0.89) and 2.4 V (red, *b* = 0.96). c) Capacitive and diffusion contribution ratios at different scan rates. d) A self‐discharge curve in a half‐cell within the potential window of 1.2–3.9 V. Inset shows voltage curves before and after self‐discharge test. e) GITT curves at 50 mA g^−1^ and the calculated ionic diffusion coefficient. f) The impedance plots of Fe‐HATNTA before and after 500 cycles at 5 A g^−1^. g) Schematic of the Li^+^ ions diffusion.

To unveil the origin of superior rate performance of Fe‐HATNTA, the charge storage behaviors are investigated using CV measurements at different scan rates (**Figure** **4**a). The current (*i*) as a function of the scan rate (*v*) is used to determine *b*‐values via a following power‐law relationship^[^ ^17^ ^]^ *i = av^b^*, where *a* and *b* are adjustable coefficients, with *b*‐values deduced from the slope of the linear fit between log *i* and log *v*. Figure S17, Supporting Information, shows the good linear fit between log *i* and log *v*, and the calculated *b*‐values at different operating potential vary from 0.89 to 0.96 (Figure 4b). High *b*‐value indicates that capacitive charge storage is dominant in the hybrid diffusion and capacitive electrochemical behavior of Fe‐HATNTA. Moreover, the contribution of capacitive and diffusion‐controlled process can be quantitatively separated based on the equation^[^ ^5^ ^]^

(1) $$i=k_{1}v+k_{2}v^{1/2}$$

where *k*_1_ *ν* and *k*_2_ *ν* are on behalf of the surface capacitive and the diffusion‐controlled contributions, respectively. Notably, the capacitive contribution is ramped from 72% to 88% with the increase of the scan rate, indicating a high capacitive charge storage (Figure 4c).

Considering the pseudocapacitive charge storage mechanism, anti‐self‐discharge performance needed to be further evaluated using capacity retention (retained/initial capacity).^[^ ^45^ ^]^ Figure 4d shows the self‐discharge curves, in which the cell is fully charged to 3.9 V followed by resting 5 days, and then the retained discharge capacity is tested. The corresponding capacity retention after self‐discharge behavior remained at 86% with a discharge capacity of 213 mAh g^−1^ delivered, demonstrating a high stability in the fully charged state. It can be concluded that Li^+^ ions bonded with active sites are difficult to self‐diffuse, contributing to the anti‐self‐discharge property.

Additionally, the intermittent titration technique (GITT) technique is further carried out to explore the ion‐diffusion kinetics. The average of Li^+^ ions diffusion coefficient (*D*_Li_^+^) in Fe‐HATNTA is calculated to be 1.53 × 10^−9^ and 1.27 × 10^−9^ cm^2^ s^−1^ during the discharge and charge phase (Figure 4e), respectively, which surpassing HATNTA (Figure S18, Supporting Information) and many other organic cathodes (Table S1, Supporting Information), evidencing the superior ions transport ability. The electrochemical impedance spectroscopy (EIS) measurement is simultaneously conducted in Figure 4f. Fe‐HATNTA exhibits the charge‐transfer resistance (*R*_ct_) of 60 Ω before cycling and 20 Ω after cycling, while HATNTA reaches to 370 Ω before and after cycles (Figure S19, Supporting Information), indicating the essentially enhanced electron transport in the MOCP. The reduced *R*_ct_ of Fe‐HATNTA after 500 cycles also suggests the improved electrode‐electrolyte wetting and interface, responsible for the ionic conduction and excellent rate performance.^[^ ^46^ ^]^

Based on the discussions above, a schematic illustration on ionic diffusion is proposed (Figure 4g). This Fe‐HATNTA tube with hierarchical porous structure ensures multiple transport pathways for ions and abundant contact interface of electrolyte with active materials, providing more exposed active sites and facilitating the conduction of Li^+^ ions. Moreover, the capacitive‐controlled characteristic of Fe‐HATNTA also favors the rapid ions transfer and charge storage, responsible for the fast reaction kinetics. Ultimately, a favorable combination is constructed for facilitating the lithiation/delithiation reactions of Fe‐HATNTA, thereby achieving an excellent rate capability and high specific capacity, and further underscoring the significance of good ion transport on charge storage.

### 2.5 Density Functional Theory Theoretical Analysis

**Figure 5:**
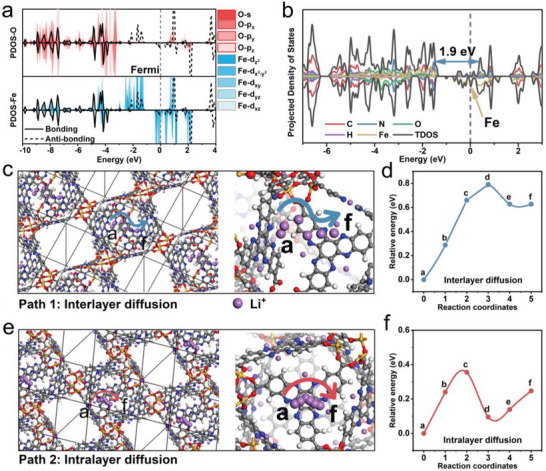
DFT theoretical analysis. a) The PDOS and COHP diagram for Fe‐HATNTA monomer. Red‐white peaks represent the s orbital and the three p orbitals of O; blue‐white peaks represent the five d orbitals of Fe; and the black line represents the bonding orbital (black solid line) and antibonding orbital (black dashed line) of Fe and O; the gray short dash at 0 eV is the Fermi level. b) The PDOS and TDOS of Fe‐HATNTA. The blue double arrow indicates that the band gap is 1.9 eV when there is no Fe—O peak. c) Interlayer diffusion path diagram of Li^+^ ions, from the “a” site to the “f” site. d) Interlayer diffusion energy barrier diagram of Li^+^ ions. e) Intralayer diffusion path diagram of Li^+^ ions, from the “a” site to the “f” site. f) Intralayer diffusion energy barrier diagram of Li^+^ ions.

Density functional theory (DFT) calculations are used to deeply delve into the intrinsic electronic structure and ionic conduction mechanism of Fe‐HATNTA. The combinations of the crystal orbital hamilton populations (COHP) theory^[^ ^47^ ^]^ and projected density of states (PDOS) diagram (**Figure** **5**a) are used to investigate the coordination bonds between Fe and O in Fe‐HATNTA. It can be easily found that Fe is coordinated with O due to the clear COHP line, and coincides with some of the PDOS peak.^[^ ^48^ ^]^ All the bonding orbitals are below the Fermi level, indicating their strong and stable coordination bonds between Fe ions and HATNTA.^[^ ^49^, ^50^ ^]^

Subsequently, the electronic structure of Fe‐HATNTA is calculated in detail to obtained its PDOS and TDOS (Total Density of States) diagram (Figure 5b). Interestingly, there is an obvious density of electronic states peak on the Fermi level, which mainly contributed by Fe element, leading to a much narrower LUMO–HOMO gap (the band gap of 1.9eV in HATNTA almost disappears) due to the coordination between Fe and O. This underscores the potential in fast charge‐transfer, and is consistent with the extremely small *R*_ct_ (Figure 4f).

Furthermore, the energy barriers of Li^+^ ions diffusion are calculated to evaluate the difficulty of ionic migration within the framework. The diffusion barriers of Li^+^ ions along different paths are obtained by transition state search of climbing image‐nudged elastic band^[^ ^51^ ^]^ in DFT calculation (Figure 5 and Movies S1 and S3, Supporting Information). The N atoms are chosen as the binding sites for the Li^+^ ions diffusion, since pyrazine N is the only redox active site bonded to lithium in the electrochemical process, which has also been corroborated by the previous report,^[^ ^7^ ^]^ and the calculation results of the charge density difference diagram (Figures S20 and S21, Supporting Information). Two representative directions of ionic transfer paths are displayed in Figure 5c,e, namely the interlayer (Path 1) and intralayer (Path 2) diffusion from the “a” site to the “f” site. As a result, the diffusion energy barriers along each path are impressively below 0.78 eV (interlayer diffusion) and 0.36 eV (intralayer diffusion) (Figure 5d,f), favoring fast ionic conduction. In contrast, the intralayer diffusion barrier of HATNTA is as high as 1.79 eV (Figure S22, Supporting Information). The much larger diffusion barrier of HATNTA is unfavorable to the diffusion of Li^+^ ions. The small diffusion barrier of Fe‐HATNTA can be attributed to the inherent features, such as the reduced distance of ionic diffusion, and abundant Li^+^ ions‐affinity building unit. Accordingly, Fe‐HATNTA is awarded the excellent electrochemical performance at high rates.

## 3 Conclusions

In conclusion, Fe‐HATNTA MOCP tubes with hierarchical porous structure are successfully synthesized using HATNTA as the self‐engaged template to coordinate with Fe^2+^ ions. Such a hierarchical tube ensures abundant contact between electrolyte and active sites, shortens the diffusion distance, and provides more favorable transport pathways for Li^+^ ions. When it is used as the cathode material for LIB, Fe‐HATNTA MOCP delivers high specific capacity (244 mAh g^−1^ at 50 mA g^−1^, 91% of theoretical capacity), excellent rate capability (128 mAh g^−1^ at 9 A g^−1^), and a long‐term cycle life (73.9% retention over 3000 cycles at 5 A g^−1^).

To further disclose the reasons for the glaringly high electrochemical performance, the comprehensive investigations are conducted. XPS and in situ Raman spectra are employed to track the reversible redox reaction during the charging/discharging processes. Kinetic analyses confirm a faster, pseudo‐capitative dominated redox reactions, a high diffusion coefficient (the overall *D*_Li_^+^ of 10^−9^ cm^2^ s^−1^), and low interfacial resistance. Moreover, DFT calculations further confirm the strong coordination bonds between Fe and O, and indicate a much‐narrowed LUMO–HOMO gap, as well as the low energy barriers for Li^+^ ions migration along both interlayer and intralayer diffusion paths in Fe‐HATNTA.

This work is considerably promising for the superior performance MOCPs‐based cathode materials by constructing the hierarchical porous structure. We also envision that the ionic and electronic conduction of such MOCPs can be feasibly tuned by some of appropriate design, such as rationally choosing the monomers and metal cations, as well as regulating the morphologies and pore structures.

## 4 Experimental Section

### Materials and Chemicals

Hexaketocyclohexane octahydrate was purchased from Sigma‐Aldrich. Ferrous chloride tetrahydrate and 3,4‐diaminobenzoic acid were purchased from Aladdin Reagent Corp. Ethanol and dimethylacetamide (DMAc) were obtained from Sinopharm Chemical Reagent Co., Ltd. All reagents were of analytical grade, and used without further purification.

### Synthesis of Hexaazatrinaphthalene Tricarboxylic Acid

HATNTA was synthesized according to a previous report.^[^ ^33^ ^]^ A mixture of hexaketocyclohexane octahydrate (1.57 g, 5 mmol) and 3,4‐diaminobenzoic acid (2.51 g, 16.5 mmol) were dissolved in 100 mL acetic acid. Then, this solution was heated to 150 °C under reflux for 5 h. The dark green suspension was cooled and then filtered and washed with water, ethanol, and acetone to obtain HATNTA (2.50 g, the yield was about 97%).

### Synthesis of Iron‐Hexaazatrinaphthalene Tricarboxylic Acid Coordination Polymer

HATNTA (51.6 mg, 0.1 mmol), ferrous chloride tetrahydrate (198 mg, 1 mmol) and 1 mL 1 m hydrochloric acid were added in 10 mL DMAc to undergo a solvothermal reaction without stirring at 120 °C for 48 h. After cooling down, the dark brown precipitation was separated through centrifugation and successively washed with DMAc and ethanol for several times, then was dried at 80 °C under vacuum for 24 h.

### Material Characterizations

PXRD pattern was performed on a Bruker D8 Advance diffractometer (Cu K*α*‐radiation) operating at 40 kV and 20 mA in the range of 10°–60° with sweep rate of 0.5° min^−1^. BET was carried out using a Quantachrome Autosorb‐IQ2 to analyze the surface area and pore size distribution. FESEM was conducted on a JEOL JSM‐6700F, equipped with the EDS to analyze the elemental distributions. TEM was taken on a JEOL JEM‐2100F. FTIR spectroscopy was obtained on a Thermal Nicolet380 spectrometer from 500 to 4000 cm^−1^. The Raman spectra were recorded using a LabRAM HR Evolution (Horiba, France) with a 532 nm laser. XPS measurements were carried out on ESCALAB 250Xi with Al‐K*α* source (1486.6 eV) as X‐ray source.

### The Theoretical Capacity Calculation

The theoretical capacity can be calculated based on the following equation:

(2) $$C_{\mathrm{theor}}=\frac{nF}{3.6\times M}$$

where *n* is the number of electrons transferred per repeat unit. The Faraday constant F is equal to 96485 C mol^−1^, and *M* is the molar weight of each repeat unit.

The molecular weight of each repeat unit in Fe‐HATNTA was 600 g mol^−1^. The 6 electrons were transferred in each repeat unit. Hence, the theoretical capacity of Fe‐HATNTA was calculated to be 268 mAh g^−1^.

### Electrochemical Measurements

For the preparation of Fe‐HATNTA cathode, a slurry mixture was prepared by mixing active material, carbon black, and polyvinylidene fluoride binder in the ratio of 6:3:1 or 8:1:1 in *N*‐methyl‐2‐pyrrolidone. The resulting slurry was spread onto a piece of aluminum foil and dried at 80 °C for 12 h under vacuum. Afterward, the coated aluminum foil was punched into circle with a diameter of 12 mm. The mass loading of active material in each disk was about 0.8 mg cm^−2^. The CR2032 coin‐type cell was assembled by using Fe‐HATNTA cathode, lithium foil as the anode, Celgard 2400 polypropylene membrane as the separator, and 1.0 mol L^−1^ LiTFSI‐DOL/DME solution as the electrolyte. The electrochemical tests were carried out using a LAND‐CT 2001A battery tester (Wuhan LAND Electric Co., Ltd.). CV tests were performed on the AutoLab electrochemical workstation in a voltage range of 1.2–3.9 V. EIS was conducted on an AutoLab electrochemical work station in the frequency of 0.01–1000 kHz at a 10 mV AC amplitude. For the ex situ XPS measurements, the coin cells at different charge states were disassembled in an Ar‐filled glovebox, and then the cycled cathodes were washed with DME solvent several times to remove residual electrolytes before drying at 80 °C in a vacuum. In situ Raman measurements were made in the stainless‐steel cell cavity with a quartz window (Beijing Science Star Technology Co. Ltd). The cathode slurry (6:3:1) was spread onto the aluminum grid, and then dried at 80 °C for 12 h. The excitation source was the laser at 532 nm (LabRAM, HR Evolution). The spectra of cells were collected per 120 s during the CV cycling process at 0.5 mV s^−1^ in the voltage range of 1.2–3.9 V. GITT was applied with a 30 min current pulse, followed by relaxation time of 1 h.

### Density Functional Theory Calculations

DFT calculations were performed through the projector augmented wave method by using the Vienna ab initio simulation package VASP. The generalized gradient approximation method with the Perdew‐Burke‐Ernzerhof was adopted as the exchange‐correlation functional. The kinetic cutoff energy was set to 400 eV. The k‐mesh in Brillouin zones was determined based on Monkhorst–Pack k‐point grids. All the calculation uses gamma k‐points and the convergence tolerance for the residual force and energy on each atom during structure relaxation were set to 0.05 eVÅ^−1^ and 10^−5^ eV. Spin polarization was considered in all calculations. To simulate the lifetime of metal DOS and make the DOS smoother, we used Gaussian smearing and set the sigma is 0.05 eV, and scatter 2000 points for calculation DOS. The optimization of Fe‐HATNTA monomer cell parameters was carried out by the method of fixing the lattice vector (modify constr_cell_relax file in VASP and recompile). Fe‐HATNTA and HATNTA 2D cell (Used to evaluate Fe—O bond) parameters were obtained as 32.918 × 32.918 × 20.000, *a* = *b* = 90°, *c* = 120°. Fe‐HATNTA cell parameters were obtained as 26.008 × 26.008 × 26.481, *a* = *b* = *c* = 90°. When calculating the diffusion energy barrier by the NEB method, due to the large number of atoms (421 atoms), some atoms are fixed to facilitate the calculation of convergence. The Lobster software^[^ ^52^ ^]^ was used to performed the COHP analysis,^[^ ^47^ ^]^ for the bonding and anti‐bonding information in the d orbital.

## Conflict of Interest

The authors declare no conflict of interest.

## Author Contributions

Y.W. and Z.Q. contributed equally to this work. Y.W. carried out the experiments. Z.Q., D.C., and S.W. performed computational calculations. K.L. performed the TEM measurements. L.Y., Z.W., and Y.Z. performed characterizations. S.Y. conceived the project. S.Y., Z.W., Y.L., and L.S. were responsible for the founds and platform. Y.W. and Z.Q. prepared the manuscript. All authors discussed and revised the manuscript.

## Supporting information

Supporting Information

Supplemental Movie 1

Supplemental Movie 2

## Data Availability

The data that support the findings of this study are available from the corresponding author upon reasonable request.
