# Supplementary material for: High‐Rate Organic Cathode Constructed by Iron‐Hexaazatrinaphthalene Tricarboxylic Acid Coordination Polymer for Li‐Ion Batteries
Source: Adv Sci (Weinh). 2022 Nov 10;9(36):2205069. doi: 10.1002/advs.202205069 (PMC9798962; doi:10.1002/advs.202205069)
Supplement: Supplementary file 1 — Supporting Information [file ADVS-9-2205069-s003.pdf]

Supporting Information

High-Rate Organic Cathode Constructed by Iron-Hexaazatrinaphthalene Tricarboxylic Acid Coordination Polymer for Li-Ion Batteries

Yifan Wang[1,2], Zelong Qiao[3], Kexin Liu[1,2], Le Yu[3], Yingying Lv[2], Liyi Shi[2,4], Yin Zhao[2], Dapeng Cao[3], Zhuyi Wang[2], Shitao Wang[3]*, Shuai Yuan[2]**

[1]School of Materials Science and Engineering, Shanghai University, Shanghai 200444, China

[2]Research Centre of Nanoscience and Nanotechnology, Shanghai University, Shanghai 200444, China

[3]State Key Lab of Organic-Inorganic Composites, Beijing University of Chemical Technology, Beijing 100029, China.

[4]Emerging Industries Institute, Shanghai University, Jiaxing 314006, Zhejiang, China

*Correspondence and requests for materials should be addressed to: Z. Wang (email: bamboo2009@shu.edu.cn); S. Wang (email: stwang@buct.edu.cn); S. Yuan (email: s.yuan@shu.edu.cn).

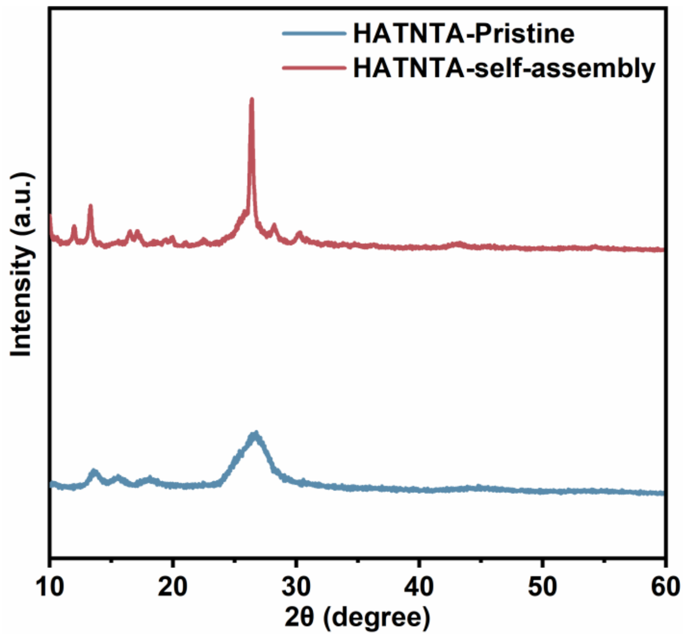

Figure S1. PXRD patterns of HATNTA and self-assembled HATNTA.

Compared to Fe-HATNTA, PXRD patterns of HATNTA and self-assembled HATNTA show the distinct difference in structure.

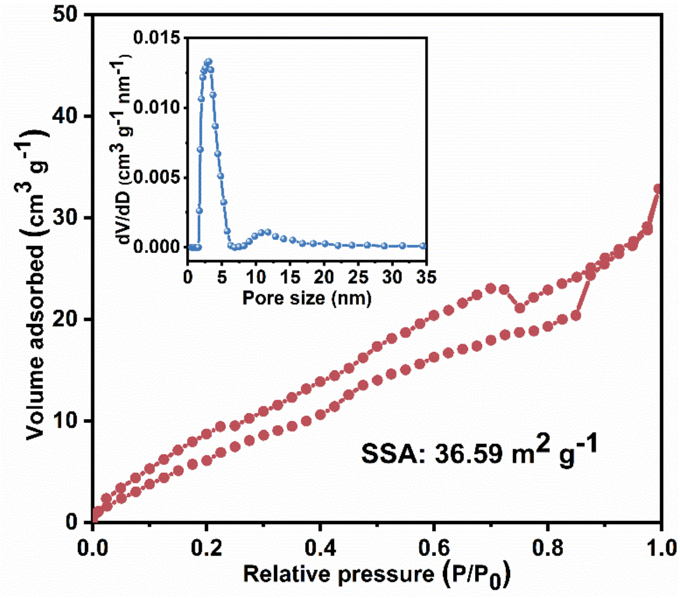

Figure S2. N_2 sorption isotherm of HATNTA. Inset: Pore size distribution obtained from the N_2 isotherm.

The specific surface area of HATNTA is about 36.59 m^2 g^{-1} with the insufficient pores, indicating the poor ions transport and limited accessible active sites.

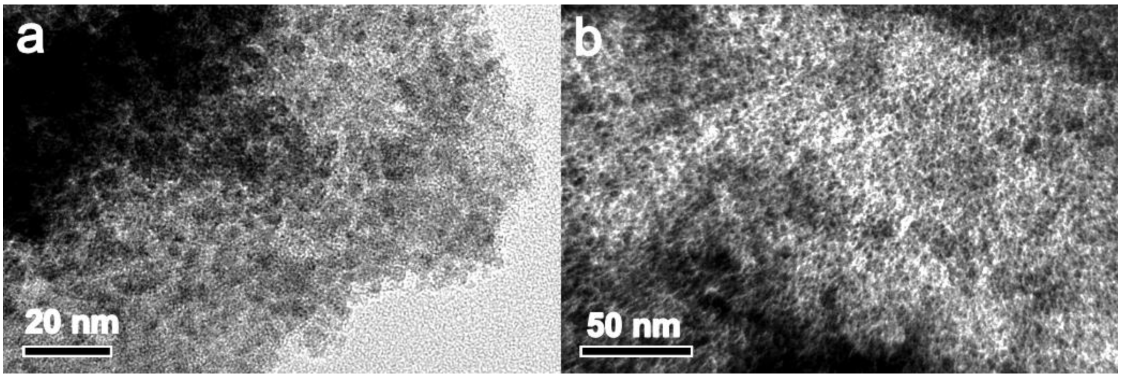

Figure S3. The HRTEM images of Fe-HATNTA. a) scale bar: 20 nm. b) scale bar: 50 nm.

HRTEM images further confirm the numerous mesopores, which are constructed by accumulation of crystals, providing an alternative for Li^+ ion transport and shortening Li^+ ion diffusion distance.

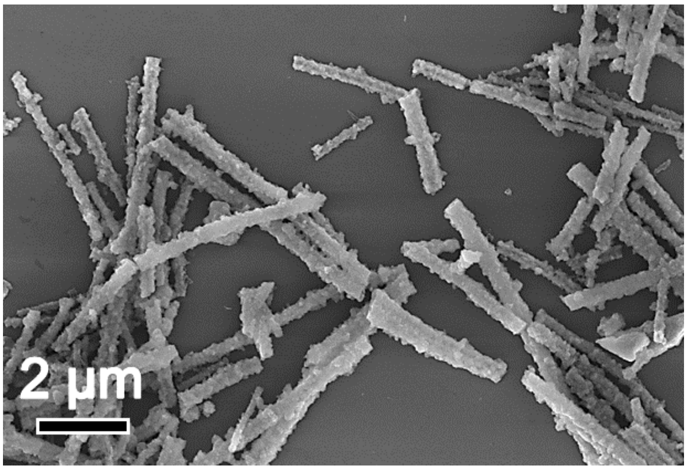

Figure S4. The SEM images of Fe-HATNTA.

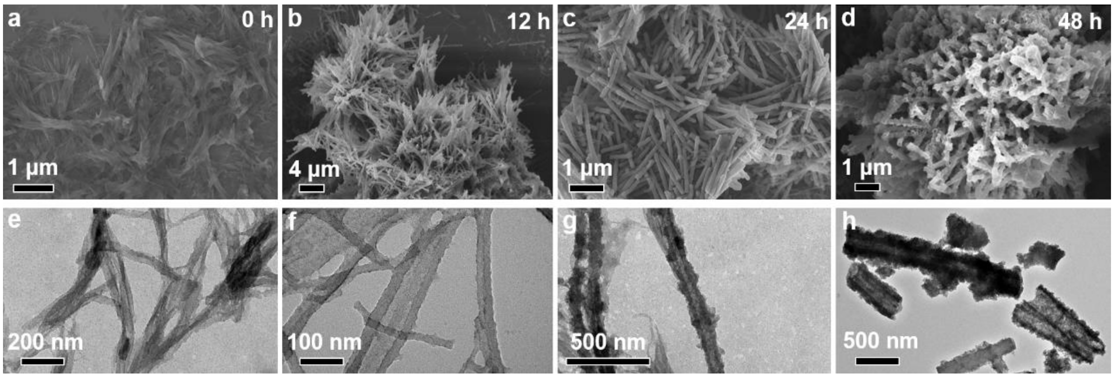

Figure S5. The corresponding morphologies of the obtained products recorded at different reaction stages: FESEM and TEM images of (a, e) 0h, (b, f) 12h, (c, g) 24h, and (d, h) 48h.

The formation mechanism of the tube-like nanostructure is further revealed by investigating the growth process. The varied morphologies at different reaction stages are characterized via FESEM and TEM observations (Figure S5). At the initial stage of the reaction, driven by a combination of the non-covalent interactions such as hydrogen bonding and π-π stacking in the solution, HATNTA molecules assemble into a fibrous structure (Figure S5a, e). As the reaction time is further prolonged, iron ions begin to coordinate with HTANTA to form Fe-HATNTA nanocrystals epitaxially growing on the surface of HATNTA nanorod assemblies (Figure S5b, f**)**. In this stage, Fe-HATNTA nanocrystals keep the growth on the surface of HATNTA nanorod-shaped assemblies, while HATNTA rod-shaped assemblies are continuously consumed (Figure S5c, g). At the end of the reaction, the rod-shaped HATNTA reactant is completely consumed to generate hierarchical Fe-HATNTA nanotubes consisting of numerous nanoparticles (Figure S5d, h).

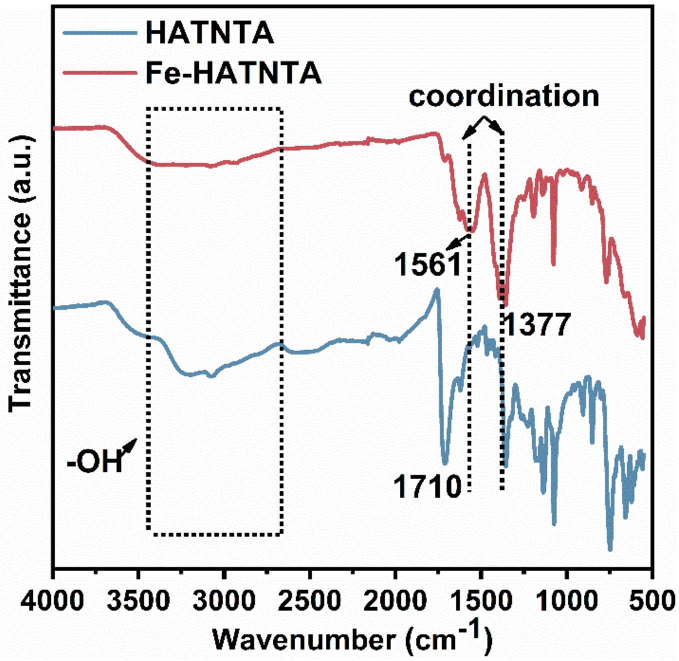

Figure S6. FT-IR spectra for Fe-HATNTA and HATNTA ligand.

As shown in Figure S6, the signal at 3400-2700 cm^{-1} is contributed from the stretching vibration of O-H bond, which is clearly weakened in Fe-HATNTA. The absorption peak at 1710 cm^{-1}, ascribed to the stretching vibrations of anti-symmetrical $-COO^-$ groups, shifts to 1561 cm^{-1}, and symmetrical stretching vibrations of $-COO^-$ groups at 1377 cm^{-1} are unambiguously strengthened. These results indicate the successful coordination of iron ions and HATNTA.

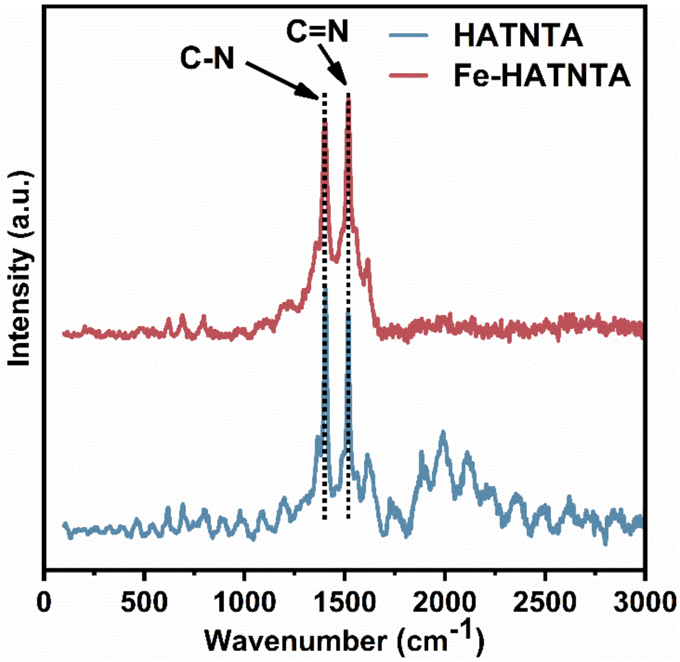

Figure S7. Raman spectra of Fe-HATNTA and HATNTA.

The two characteristic stretching vibrations of C=N (1520 cm^{-1}) and C-N (1403 cm^{-1}) are still preserved in the Raman spectrum of Fe-HATNTA, indicating that only carboxyl groups are coordinated with iron ions.

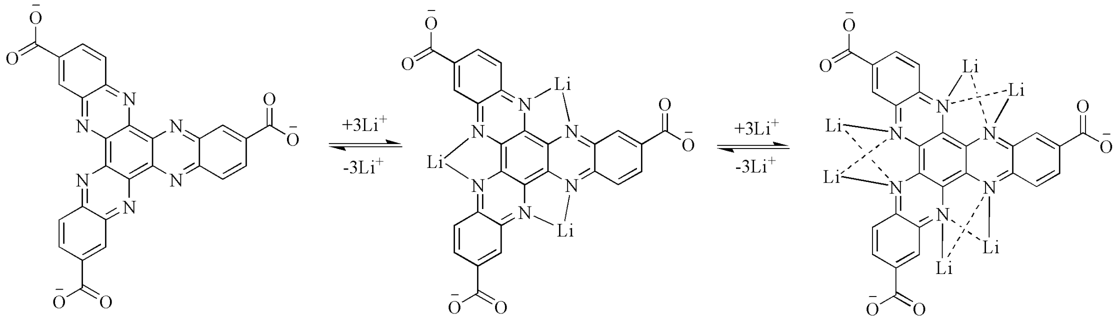

Figure S8. The proposed two-step three-electron transfer mechanism in Fe-HATNTA.

The corresponding reactions of the redox peaks are in accordance with the proposed two-step three-electron transfer mechanism for HATN-based derivatives, as shown in Figure S8. Therefore, the Fe-HTATNTA exhibited two redox couples (2.42 V/2.46 and 1.84 V/2.12) in cyclic voltammetry (CV) curves.

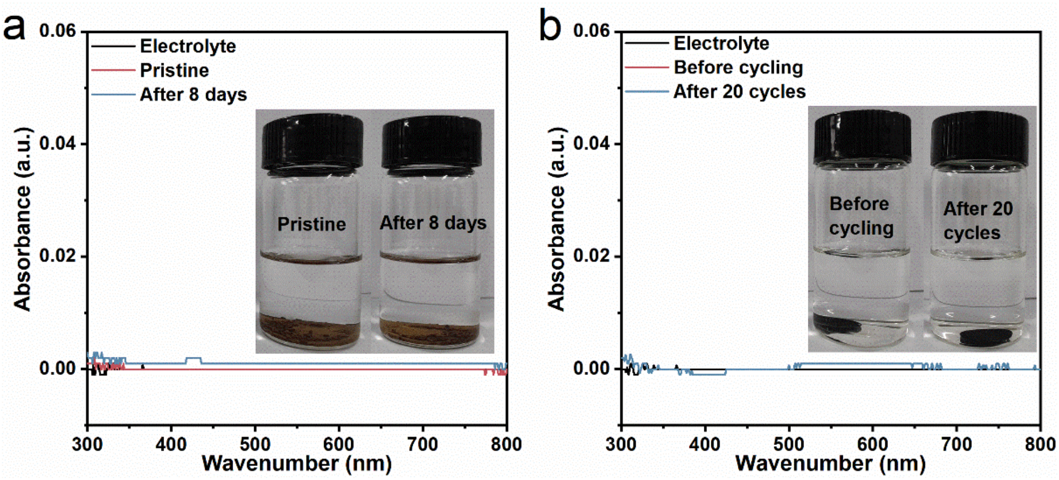

Figure S9. Dissolution test for Fe-HATNTA. a) UV/ vis spectra of Fe-HATNTA powder after 8 days. b) UV/vis spectra of Fe-HATNTA electrode before and after 20 cycles at 0.5 A g^{-1}.

Figure S9a shows the corresponding UV/vis spectra of Fe-HATNTA Powder after 8 days in electrolyte, and no signal is detected. The electrode after 20 cycles were also immersed in the electrolyte to further evaluate the solubility. After electrochemical reaction, the electrolyte-added Fe-HATNTA remains transparent as displayed by the photographs, and the UV-vis signal is almost undetectable (Figure S9b). The results confirm the excellent stability Fe-HATNTA in electrolyte.

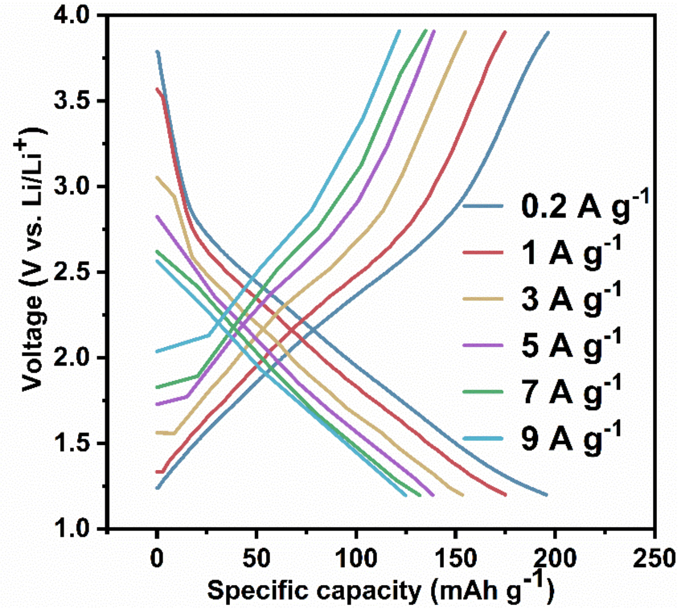

Figure S10. Charge-discharge profiles of Fe-HATNTA at various current densities.

The charge/discharge curves show the according specific capacity at current densities from 0.2 to 9 A g^{-1}. It can be easily seen the reversible specific capacity of 128 mAh g^{-1} at the high current density of 9 A g^{-1}, indicating the excellent high-rate permance.

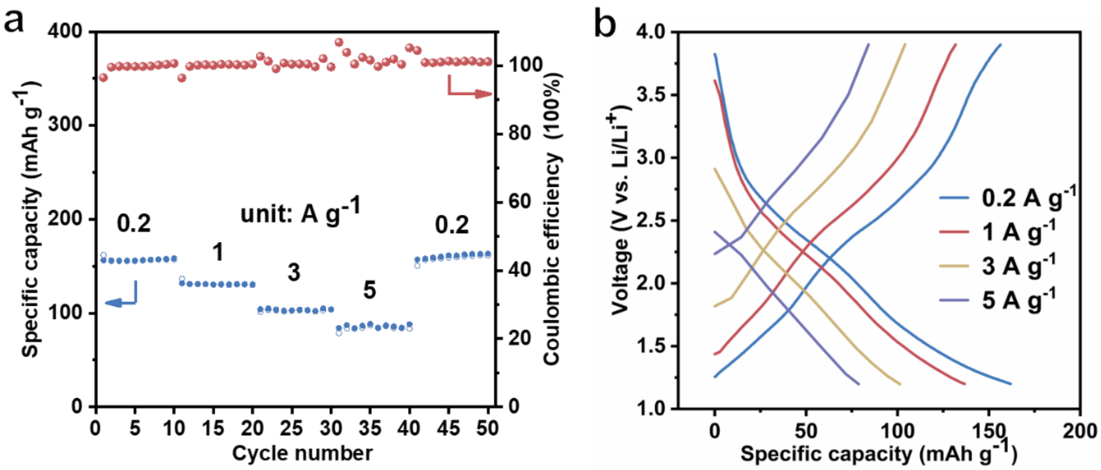

Figure S11. a) Rate performance of HATNTA at different current densities. b) Charge-discharge profiles of HATNTA at different current densities.

Figure S11 shows capacities of 156.3, 131.8, 104.1, and 84.1 mAh g^{-1} at according current rates of 0.2, 1, 3, and 5A g^{-1}, indicating an inferior rate performance of HATNTA ligand.

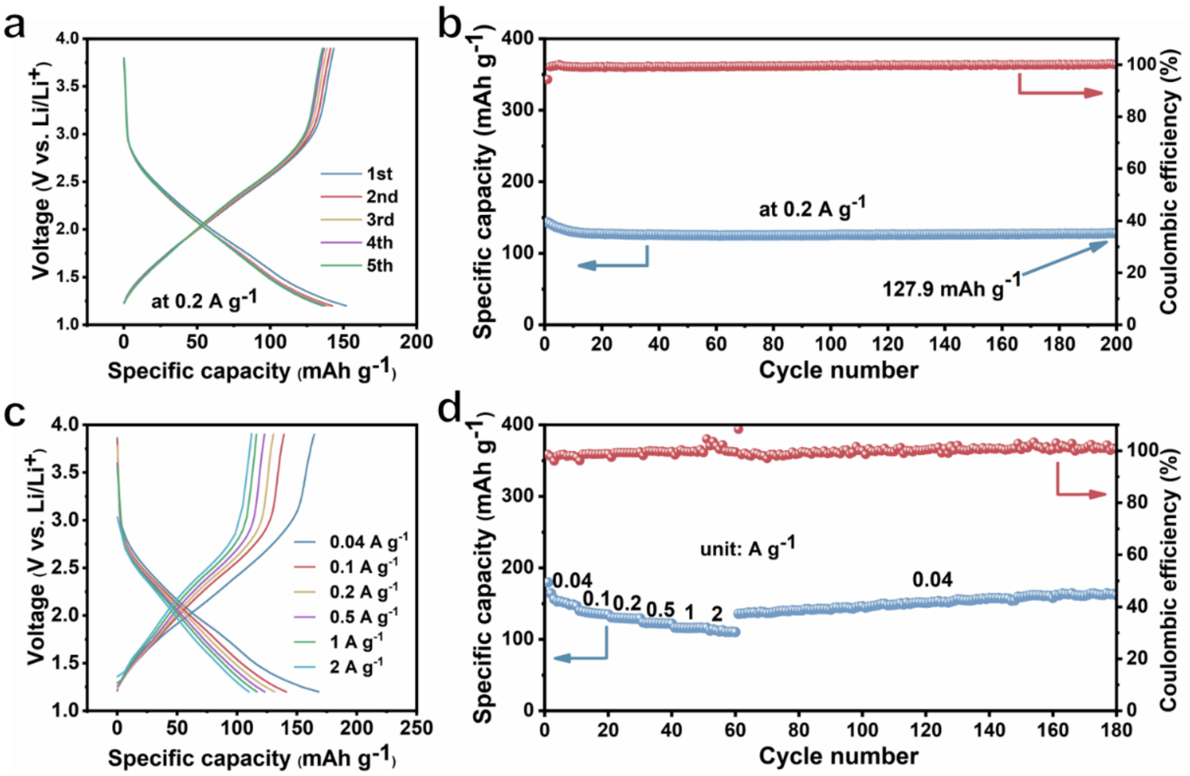

Figure S12. Electrochemical performance of Fe-HATNTA in the mass ratio of 8:1:1 (active materials: carbon black: polyvinylidene fluoride). a) Charge–discharge curves at 0.2 A g^{-1}. b) Cycling performance for at a current density of 0.2 A g^{-1}. c-d) Rate performance at various current densities.

Additionally, this MOCPs system is rendered electrochemically brilliant with the high ration of active materials (active material: carbon black: polyvinylidene fluoride = 8:1:1). When cycled at 200 mA g^{-1}, the capacity still sustains at 127.9 mAh g^{-1} over 200 cycles, yielding a capacity retention of 89.3% (Figure S12b). The corresponding charge/discharge curves are highly overlapped as shown in Figure S12a. It should be mentioned that the rate capability is also striking in this high ratio of active material, demonstrating a high capacity of 112.1 mAh g^{-1} at a high loading current of 2 A g^{-1} (Figure S12c, d). When the current density is reverted to 40 mA g^{-1}, the specific capacity also gradually recovers to its initial level of 161.6 mAh g^{-1}.

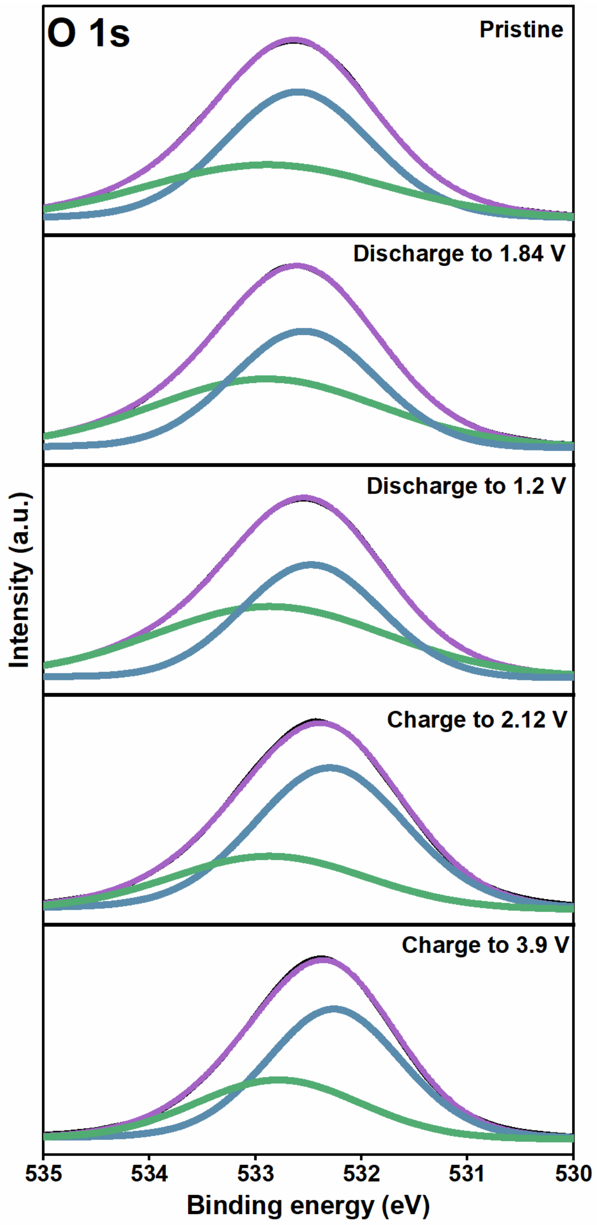

Figure S13. High-resolution O 1s spectra of Fe- HATNTA at different electrochemical states.

As shown in Figure S13, the O 1s deconvolution produced the peaks centered approximately at 532.7 and 532.1, which are separately assigned to C-O and C=O, and there is no obvious change occurred in the different charge state, pointing to the noninvolvement of COO^- groups for redox reaction.

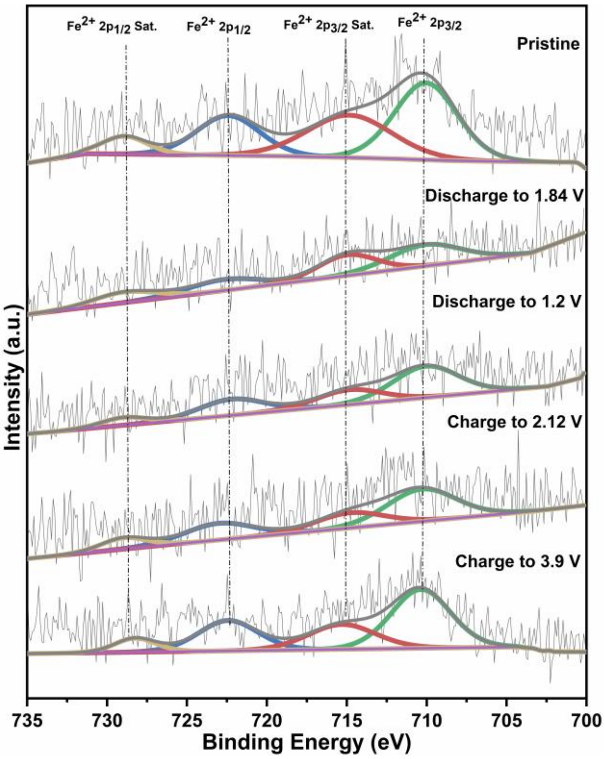

Figure S14. High-resolution Fe 2p spectra of Fe- HATNTA at different electrochemical states.

In addition, in the Fe 2p XPS spectra (Figure S14), the deconvoluted peaks at 709.9 eV and 715.8 eV are attributed to Fe $2p_{3/2}$ and its satellite, respectively. At the same time, the peaks displayed negligible change in cycling process, indicating that metal ions serve only as the bridge to stabilize Fe-HATNTA structure rather than participates in redox reaction.

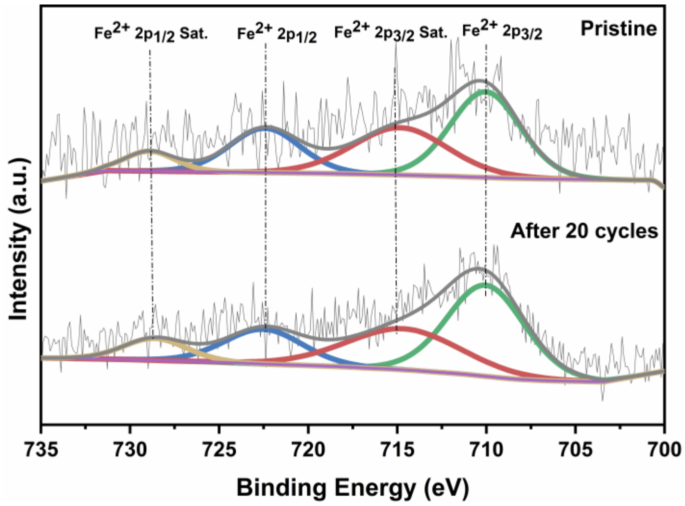

Figure S15. High-resolution Fe 2p spectra of Fe- HATNTA after 20 cycles at 0.5A g^{-1}.

After 20 cycles at 0.5A g^{-1}, there is no distinct change occurred for Fe 2p XPS spectra in Fe- HATNTA, when compared with the pristine electrode.

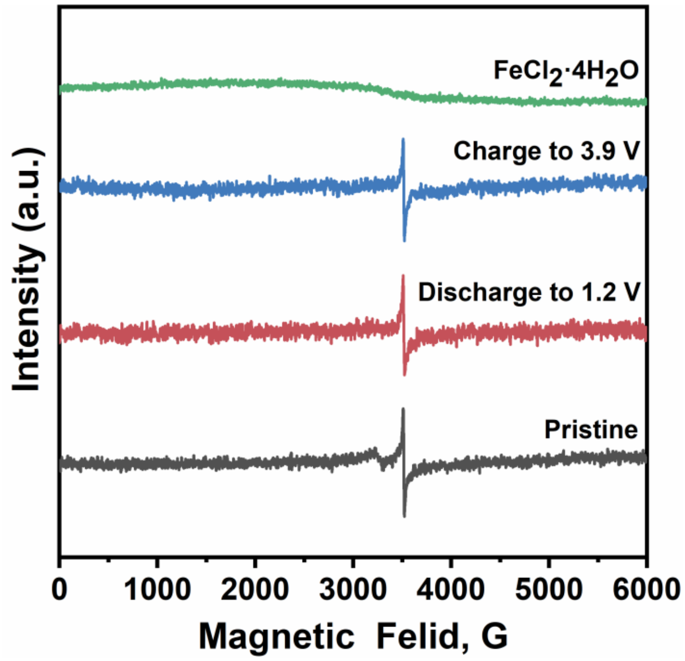

Figure S16. The EPR spectra of Fe-HATNTA in different charge state.

As can be seen from the electron paramagnetic resonance (EPR) spectra, there is no sign of Fe^{3+} ions peak in the different charge states, indicating that Fe^{2+} ions didn't change into Fe^{3+} during the charging-discharging process.

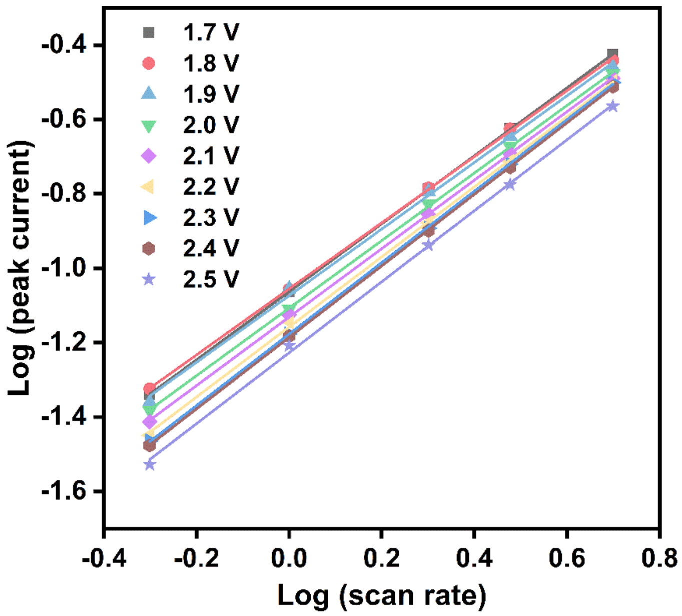

Figure S17. Log(i) versus log(v) plots in the range of 1.7-2.5 V during cathodic sweeps.

Figure S17 shows the good linear fit between log *i* and log *v*, in which *b*-values can be deduced from the slope of the plots.

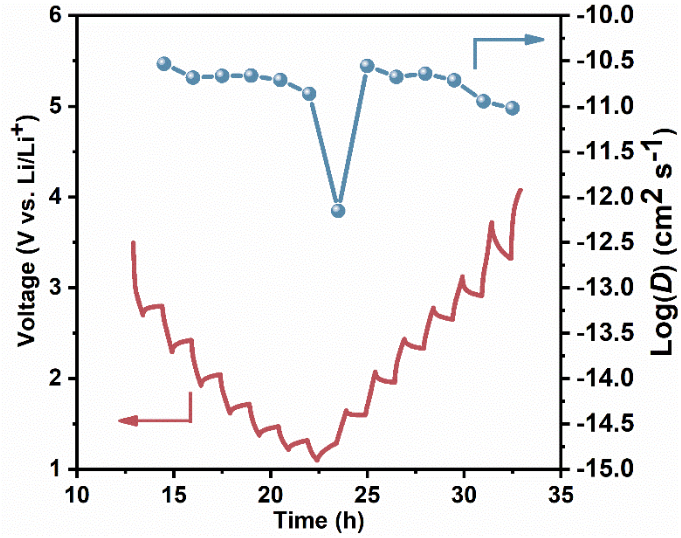

Figure S18. GITT curves of HATNTA at 50 mA g^{-1} and the calculated ionic diffusion coefficient.

The average of Li^+ ions diffusion coefficient in HATNTA is calculated to be 1.82×10^{-11} cm^2 s^{-1} and 1.87×10^{-11} cm^2 s^{-1} during the discharge and charge phase, respectively, which is lower than our Fe-HATNTA cathode.

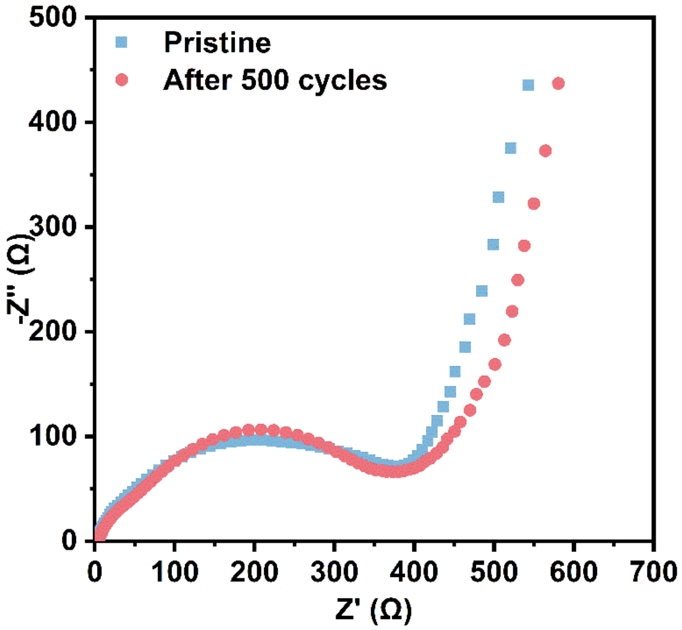

Figure S19. The impedance plots of HATNTA before and after 500 cycles at 5 A g^{-1}.

The R_{ct} of HATNTA is as high as 370 Ω before and after cycles, indicating the poor electron transport and unfavorable electrode-electrolyte contact of HATNTA cathode.

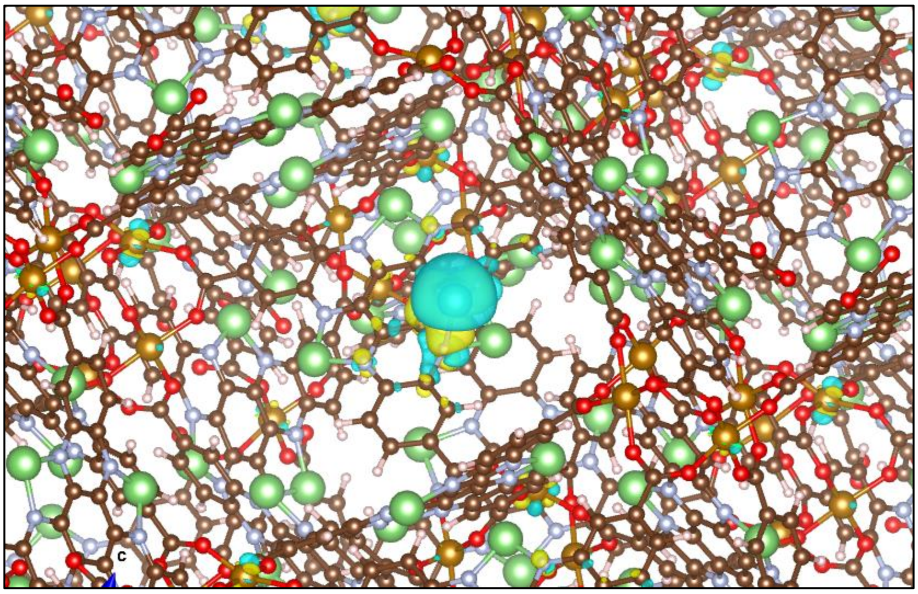

Figure S20. Differential charge density diagram of Fe-HATNTA after Li^+ ions adsorption.

Figure S20 show that the charge of Li is transferred to Fe-HATNTA, indicating that Li^+ ions are adsorbed onto the N of Fe-HATNTA.

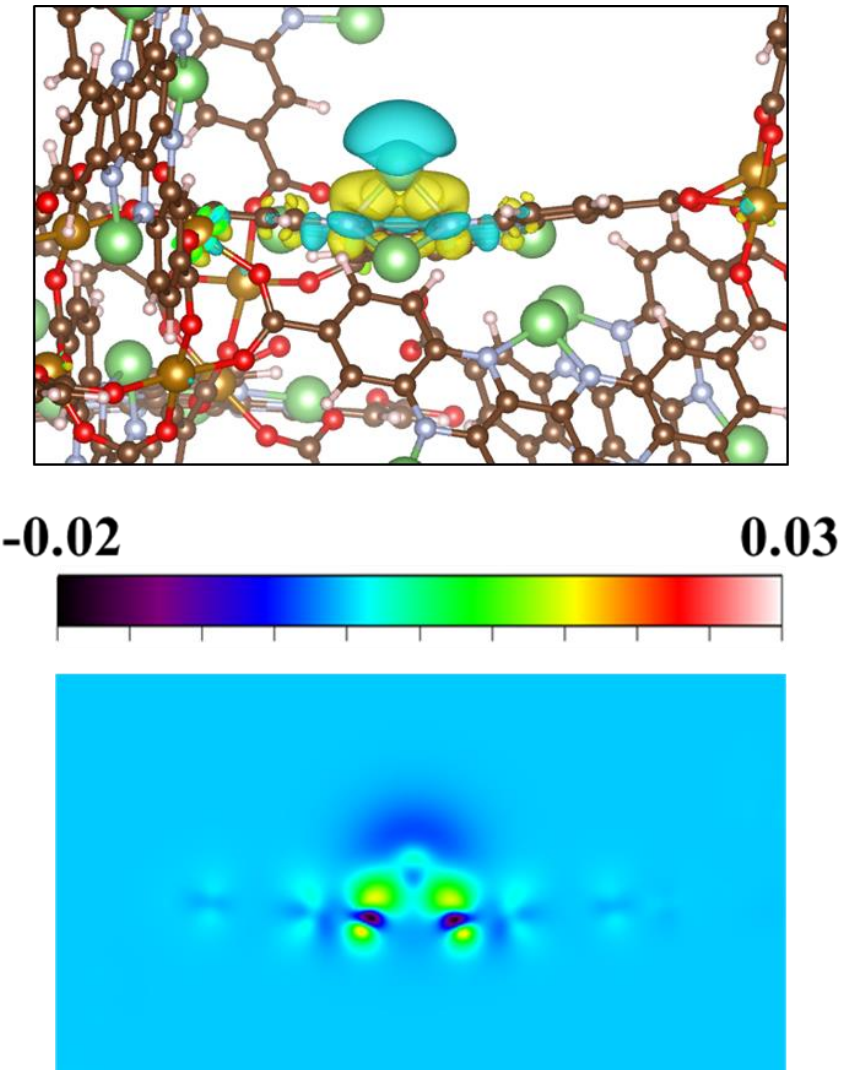

Figure S21. Main view and 2D view of Differential charge density diagram of Fe-HATNTA after Li^+ ions adsorption.

Figure S21 shows that the large amount of charges are accumulated between Li and N, thereby forming Li-N bonds.

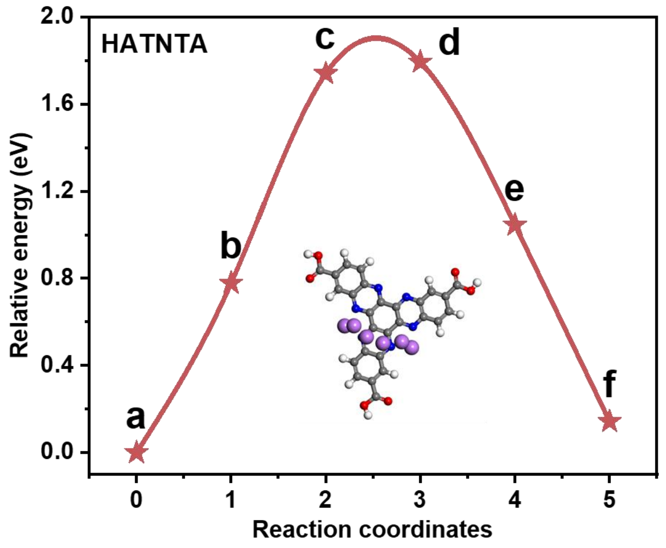

Figure S22. Diffusion energy barrier diagram of Li^{+} ions for HATNTA.

Figure S22 shows that the intralayer diffusion barrier of HATNTA is as high as 1.79 eV, which is much higher than the diffusion barrier of Fe-HATNTA (0.36 eV). The much larger diffusion barrier of HATNTA is unfavorable to the diffusion of Li+ ions, further demonstrating the advantages of our Fe-HATNTA cathode.

Table S1. Comparison of ionic diffusivities of Fe-HATNTA with reported organic and inorganic cathodes

Electrode	Ionic diffusivities ($cm^2\ s^{-1}$)	Ref.
**Fe-HATNTA**	$\sim10^{-9}$	This work
PBQDS	6×10^{-13}	[S1]
PTCDA/RGO/CNT	1.83×10^{-12}	[S2]
NTAQ	7.92×10^{-14}	[S3]
PMAQ	6.85×10^{-16}	[S3]
PAQS	1.92×10^{-11}	[42]
PIL-cat	0.28×10^{-12}	[S4]
O-Fe-MOF	1.81×10^{-11}	[41]
Co-BDBA-MOCP	1.79×10^{-14} - 2.40×10^{-14}	[S5]
Co-HIPA	$\sim10^{-13}$	[45]
$(Co, Mn)_2O_4$	1.8×10^{-15}	[S6]
Fe-doped $LiMnPO_4$@C	3.31×10^{-12}	[S7]
$H_2V_3O_8$ gradient nanowire	$0.1\sim10\times10^{-11}$	[S8]

References

[1] N.A. Tran, J.C. Leprêtre, F. Alloin, *Electrochim. Acta* **2021**, *375*, 137990.

[2] G. Zhou, Y. E. Miao, Z. Wei, L. Mo, F. Lai, Y. Wu, J. Ma, T. Liu, *Adv. Funct. Mater.* **2018**, *28*, 1804629.

[3] Z. Ba, Z. Wang, M. Luo, H. B. Li, Y. Li, T. Huang, J. Dong, Q. Zhang, X. Zhao, *ACS Appl. Mater. Interfaces* **2020**, *12*, 807.

[4] N. Patil, M. Aqil, A. Aqil, F. Ouhib, R. Marcilla, A. Minoia, R. Lazzaroni, C. Jérôme, C. Detrembleur, *Chem. Mater.* **2018**, *30*, 5831.

[5] X. Tang, Y. Zhang, W. Sun, Y. Wang, *ACS Appl. Energy Mater.* **2020**, *3*, 11378.

[6] B. Wu, Y. Xie, Y. Meng, C. Qian, Y. Chen, A. Yuan, X. Guo, H. Yang, S. Wan, S. Lin, *J. Mater. Chem. A* **2019**, *7*, 6149.

[7] H. Yang, C. Fu, Y. Sun, L. Wang, T. J. C. Liu, *Carbon* **2020**, *158*, 102.

[8] Y. Cheng, J. Shu, L. Xu, Y. Xia, L. Du, G. Zhang, L. Mai, *Adv. Energy Mater.* **2021**, *11*, 2100026.
